# Cauterization of Variolous Pustules

**Published:** 1826-07

**Authors:** 


4. *Variolous Pustules.* A good deal of discussion has lately taken place in France relative to the power of caustic in preventing the formation of pustules and the disfiguring cicatrices so often left after severe small-pox. Messrs. Velpeau and Bretanneau have strongly advocated the measure; but, it is always best to wait till others, besides the original discoverers, have tried the efficacy of any new procedure, before we place much confidence in their statements. It now appears that considerable abatement must be made from the good effects of cauterization, as first proclaimed by the authors above-mentioned. Dr. Meyraux gave the measure a trial in the Hôpital de la Pitié, and reports that if the variolous pustules are opened with a lancet and touched with a pointed piece of lunar caustic, *on the first or second day of their appearance*, they will be annihilated, and no marks will be left—but, on the third day, it will be quite useless.—This is but small encouragement —yet it is more than was admitted during the discussions in the Institute, where it was "the opinion of several medical men who had tried the measure, that cauterization was useless in the distinct, and prejudicial in the confluent small-pox."—*Bulletin Univers.*

